# Assessing the endothelium’s role in COVID-19 severity using the HUVEC model

**DOI:** 10.3389/fimmu.2025.1689772

**Published:** 2026-01-08

**Authors:** Sanzio Silva Santana, Sètondji Cocou Modeste Alexandre Yahouédéhou, Corynne Stéphanie Ahouéfa Adanho, Jéssica Rebouças Silva, Hayna Malta Santos, Cynara Gomes Barbosa, Thassila Nogueira Pitanga, Valéria Matos Borges, Vitor Fortuna, Isa Menezes Lyra, Marilda Souza Goncalves

**Affiliations:** 1 Laboratório de Investigação em Saúde Global e Doenças Negligenciadas, Instituto Gonçalo Moniz, Salvador, Bahia, Brazil; 2 Faculdade de Biomedicina, Universidade Católica do Salvador, Salvador, Bahia, Brazil; 3 Laboratório de Pesquisa Clínica e Translacional, Instituto Gonçalo Moniz, Salvador, Bahia, Brazil; 4 Faculdade de Farmácia, Universidade Federal da Bahia, Salvador, Bahia, Brazil; 5 Departamento de Bioquímica e Biofísica, Instituto de Ciências da Saúde, Universidade Federal da Bahia, Salvador, Bahia, Brazil; 6 Programa de Pós-graduação em Imunologia, Instituto de Ciências da Saúde, Universidade Federal da Bahia, Salvador, Bahia, Brazil; 7 Departamento de Hematologia, Hospital Universitário Professor Edgard Santos, Salvador, Bahia, Brazil

**Keywords:** COVID-19 severity, endothelial dysfunction, HUVEC, cytokine storm, thromboinflammation, oxidative stress

## Abstract

**Introduction:**

Coronavirus disease 2019 (COVID-19) has been widely associated with intense systemic inflammation, endothelial injury, and a high incidence of thrombotic complications, which together contribute to disease severity and poor clinical outcomes. While endothelial dysfunction, dysregulated cytokine production, and oxidative stress are recognized features of severe COVID-19, the direct impact of circulating factors from infected individuals on endothelial cell behavior remains insufficiently characterized. Here, we examined how serum from patients with severe COVID-19 and from convalescent individuals modulates endothelial activation, inflammatory responses, and oxidative stress using human umbilical vein endothelial cells as an in vitro model.

**Methods:**

Venous blood samples were collected from individuals with severe COVID-19 (n = 13), convalescent patients (n = 11), and healthy volunteers (n = 7) during the initial phase of the COVID-19 pandemic. Human umbilical vein endothelial cells (HUVEC) were maintained in culture and exposed to 15% serum from each study group after a period of serum deprivation. The expression of genes associated with endothelial activation, thrombosis, inflammation, and oxidative stress was analyzed by quantitative real-time PCR at defined time points. In addition, the endothelial secretory profile was evaluated in cell culture supernatants using multiplex bead-based immunoassays. Statistical analyses were performed using one-way ANOVA followed by appropriate post hoc tests, receiver operating characteristic (ROC) curve analysis to assess the discriminatory capacity of biomarkers, and multivariate linear regression to identify factors associated with disease severity.

**Results and discussion:**

We investigated the role of the endothelium in modulating the cytokine storm in severe COVID-19. HUVEC were stimulated with serum from patients with severe COVID-19, convalescent individuals, and healthy volunteers. Stimulation with serum from severe cases induces significant increases in *VCAM1, F3, PROCR, IL6, IL12A, NFE2L2, HMOX1, GPX1*, and *GSR* expression within 60 minutes. Antioxidant genes SOD1 and CAT were upregulated later, after 120 minutes. HUVEC stimulated with severe COVID-19 sera showed increased levels of sICAM-1, sVCAM-1, P-selectin, sE-selectin, PECAM-1, tissue factor, thrombomodulin, and a broad range of cytokines and growth factors, such as IL-1α, IL-1Ra, IL-5, IL-6, IL-10, IL-12(p40), IL-18, IL-27, TNF-α, TGF-α, FGF-2, G-CSF, M-CSF, FLT-3L, fractalkine, eotaxin, MIG, IP-10, MIP-1β, MDC, GROa and PDGF-AB/BB. In contrast, convalescent sera induced fewer markers, specifically IL-12(p40), IL-18, FGF-2, MIP-1β, MDC, GROa, and PDGF-AB/BB, while HV sera induced significant increases in IL-12(p40), IL-27, TNF-α, VEGF, MDC, eotaxin, and GROa. ROC curve analysis revealed that P-selectin and MIP-1β levels clearly distinguish severe cases from HV. When comparing severe and convalescent groups, we observed increases in IL-27, TGF-α, sVCAM-1, IL-1α, and G-CSF levels. Furthermore, Multivariable logistic regression analysis associated disease severity with decreased IL-10 and increased MIP-1β, sICAM-1, and P-selectin.

**Conclusion:**

These findings suggest that HUVEC serves as a promising biological sensor for detecting inflammatory responses in COVID-19 patients and shows the crucial role of the endothelium in sustaining the cytokine storm that contributes to patient severity and mortality.

## 1 Introduction

Coronavirus disease 2019 (COVID-19), declared a pandemic by the World Health Organization, is caused by the SARS-CoV-2 virus and ranges from mild respiratory symptoms to severe acute respiratory distress syndrome, often resulting in high morbidity and mortality among hospitalized patients. Previous studies have indicated that approximately 15% of infected patients face a heightened risk of thromboembolic events (1, 2).

COVID-19 is characterized as an inflammatory disease marked by hypoxia, pulmonary edema, and cytokine storm, with complications intensifying in the presence of underlying conditions such as diabetes, hypertension, and atherosclerosis. These conditions can exacerbate the disease, frequently leading to multiple organ failure (3, 4).

Patients exhibiting mild COVID-19 symptoms, such as fever and cough, often do not require hospitalization unless they experience dyspnea or hypoxemia (oxygen saturation ≤94% or need for oxygenation or ventilatory support), which are indicative of severe disease states (5, 6).

SARS-CoV-2 may trigger an exacerbated immune response known as a cytokine storm, characterized by a significant rise in pro-inflammatory cytokines, such as interleukin (IL)-1 and IL-6, chemokines, and a T helper (Th) 1 response (7–9). This response can activate monocytes, induce tissue factor expression, and release other procoagulant substances, along with platelet activation (10–12). Associated endothelial dysfunction includes glycocalyx degradation and loss of the endothelium’s anti-inflammatory, antithrombotic, and permeability-regulating functions (13–15).

Endothelial damage is closely linked to increased organ failure risks in COVID-19 patients, with studies demonstrating how the virus triggers endothelial cell dysregulation, enhances pro-inflammatory cytokine production, oxidative stress, and expression of adhesion molecules such as ICAM1, VCAM1, and E-selectin (16–19). These changes lead to increased immune cell recruitment and endothelial cell activation, intensifying the inflammatory response (20–24).

Excessive activation of the endothelium and platelets, alongside an exacerbated immune response, often results from an unbalanced reaction between the infectious agent and the host, leading to oxidative system activation and contributing to COVID-19’s pathogenesis (25).

Oxidative stress is a critical factor in COVID-19’s pathogenesis, occurring when there is an imbalance between the production of reactive oxygen species (ROS) and the body’s antioxidant capacity. This imbalance can cause cellular and tissue damage, triggering inflammatory and apoptotic processes (26, 27). Key molecules, such as nuclear factor erythroid 2-related factor 2 (NFE2L2 or NRF2), glutathione, catalase, hemopexin, and superoxide dismutase, regulate this process and are vital for managing oxidative stress in severe pneumonia cases (28–32).

The interaction of endothelial cells with SARS-CoV-2 *in vivo*, particularly how they react to the virus initially present in lung epithelial cells, is still not fully elucidated. This study used human umbilical cord vein endothelial cells (HUVEC) as a model to explore their potential involvement in cytokine storm, oxidative stress, and endothelial dysfunction in patients with COVID-19.

## 2 Methods

### 2.1 Collection of samples of individuals with COVID-19 and healthy volunteers

Venous blood samples from patients with severe COVID-19 (Severe, n = 13), convalescents (Conv., n = 11), and healthy volunteers (HV, n = 7) were obtained after written consent from all study participants or their legal guardians. The protocol was conducted according to the 1975 Helsinki statement and Brazilian ethical guidelines (466-CNS-2012) and was approved by the institutional review board of the Fiocruz/Bahia Foundation (protocol number 41130920.3.0000.0040). Samples from patients with severe disease, convalescent individuals, and HV were obtained from the Intensive Care Unit (ICU) of the Salvador/BA Suburban Hospital, Irecê/BA Hospital, and Instituto Gonçalo Moniz (IGM), Salvador/BA, respectively. Severely ill patients were defined as individuals with a confirmed COVID-19 diagnosis who exhibited a severe clinical profile and required ICU admission. Convalescent individuals were defined as those who had COVID-19, were treated, and were discharged, while HV were individuals who never had COVID-19. All individuals included in this study had not received COVID-19 vaccines for, and all samples were collected during the initial period of the pandemic.

### 2.2 Cell culture

Human umbilical vein endothelial cells (HUVEC) were kindly donated by Ph.D. Ana Moretti and M.D. Heraldo Possolo de Souza, from the Faculdade de Medicina da USP/São Paulo, were cultivated in cell culture vials with a 25 cm^2^ surface area (Jet Biofil, Guangzhou, China) containing 5 mL RPMI 1640 (Gibco, New York, NY, USA) supplemented with 10% bovine fetal serum (10% SFB) heat inactivated (Gibco, New York, NY, USA) and antibiotics (100 U/mL penicillin and 10 mg/mL streptomycin) (Sigma Aldrich, St. Louis, MO, USA). Experiments were performed using cells at passages 1 to 3, and cultures were maintained in a humidified atmosphere at 37 °C with 5% CO_2_.

### 2.3 HUVEC stimulation

For the experiments, 2.5 × 10^5^ cells/well (0.38 mL) were cultivated in 24-well plates (Costa, Corning, NY, USA) for 24 h under the above conditions. After confluence, the HUVEC were deprived of RPMI 1640 (SFB 2%) for 12 h and stimulated with medium RPMI 1640 (SFB 2%) containing 15% serum from patients with severe COVID-19 (*n* = 13), convalescents (*n* = 11), and HV (*n* = 7) for different periods of time, depending on the test.

### 2.4 Gene expression assays

RNA was extracted from HUVEC after 30 min, 60 min, and 120 min of stimulation with severe, convalescent patient, and HV serum using the RNeasy Mini-Kit (Qiagen, USA), according to the manufacturer’s specifications. The concentration and purity of the extracted RNA were determined based on spectrophotometric readings using a Nanodrop 2000 (Thermo Fisher Scientific, Rockford, IL, USA) with Absorbance A260/280. Reverse cDNA synthesis (500 ng) was performed using the High-Capacity cDNA Reverse Transcription Kit (Thermo Fisher Scientific, Rockford, IL, USA), according to the manufacturer’s specifications. The synthesized cDNAs were then diluted to a concentration of 2.5 ng/µL. Real-time PCR (qRT-PCR) was performed using an ABI Prism 7500 Real-Time PCR System (Applied Biosystems, Foster City, CA, USA). To do this, mixtures containing SYBR^®^ Green PCR Master Mix plus target gene (8 μL) and cDNA (2 μL) were used. The target genes evaluated in the races were: tissue factor (*F3*), protein 1 of vascular cellular adhesion (*VCAM1*), intercellular adhesion molecule 1 (*ICAM1*), endothelin 1 (*EDN1*), endothelial protein receptor (*PROCR*), interleukin 6 (*IL6*), interleukin 12 alpha (*IL12A*), alpha tumor necrosis factor (*TNFA*), nuclear factor erythroid 2 related to factor 2 (*NFE2L2*), heme oxygenase 1 (*HMOX1*), superoxide dismutase 1 (*SOD1*), catalase (*CAT*), glutathione peroxidase 1 (*GPX1*), glutathione S-reductase (*GSR*). Glyceraldehyde-3-phosphate dehydrogenase gene (*GAPDH*) and Tubulin alpha-1C chain gene (*TUBA1C*) were used as endogenous controls for the PCR reactions. All evaluated target primers were used at a concentration of 250 pM.

### 2.5 Dosage of HUVEC supernatants stimulated with the sera

The cells were stimulated with sera from patients with severe, convalescent patients, and controls after 24 h of deprivation. Subsequently, the supernatants were collected and stored at −80 °C for subsequent analysis. The dosages were performed using the Milliplex^®^ Human Cytokine/Chemokine/Growth Factor Panel to 48 Plex Kit-Hcyta-60K-PX48 (Merck/Sigma Aldrich, USA) and Milliplex^®^ MAP Human Cardiovascular Disease Magnetic Bead Panel 2 (Merck/Sigma Aldrich, USA) according to the manufacturer’s specifications.

### 2.6 Statistical analysis

All experiments were performed in triplicates. GraphPad Prism v.6.0 (San Diego, CA, USA) and IBM SPSS Statistics v.21.0 (Armonk, NY, USA) were used for the statistical analyses. Statistical significance was set at *p <*0.05. One-way ANOVA followed by Tukey’s *post-hoc* test was applied to test the variance among multiple groups. Receiver operatoring characteristics (ROC) curve analysis was performed to evaluate the sensitivity and specificity of the biomarker concentrations in the discrimination of health conditions (severe *vs*. convalescent or severe *vs*. HV). Moreover, Multivariate Linear Regression (MLR) analyses were performed to investigate the weight of each variable, which presented a significant difference in association analyses (severe *vs*. HV) when combined.

## 3 Results

### 3.1 Serum from patients with severe COVID-19 induced significant expression of genes associated with endothelial dysfunction, thrombosis, inflammation, and antioxidant response in HUVECs

**Figure 1:**
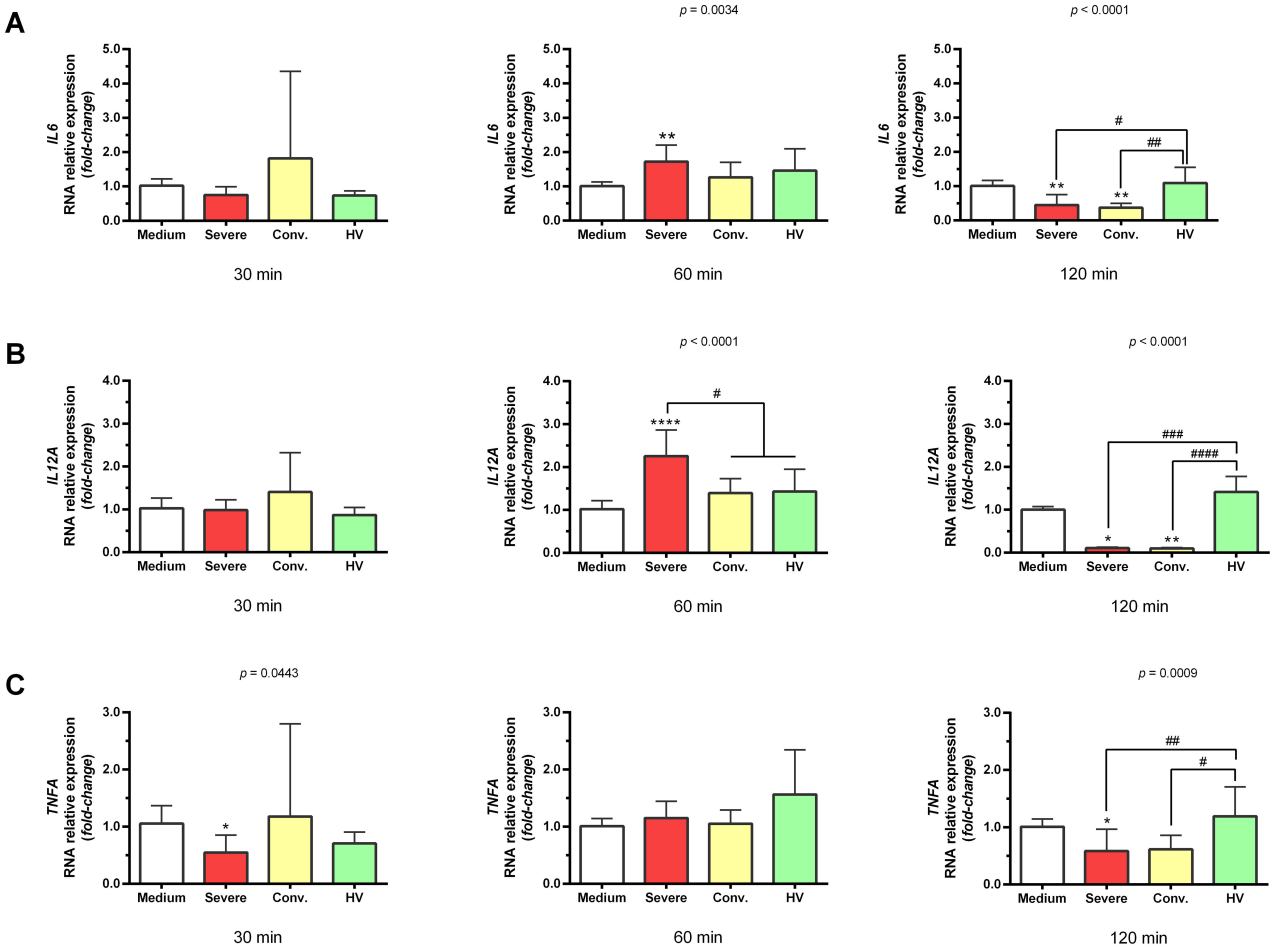
Gene expression profile related to inflammation in HUVEC stimulated with serum from severe COVID-19 patients, convalescents, and healthy volunteers (HV). HUVEC were stimulated at intervals of 30 min, 60 min, and 120 min with serum from distinct groups: severe (n = 12), convalescents (n = 11), HV (n = 7), as well as culture medium serving as the basal control (n = 6). The data presented are mean ± standard deviation. Gene expression values were determined by relative quantification using the equation: Fold-change = 2^−Δ(ΔCT)^, where ΔCT = CT_target_ − CT_endogenous_ and Δ (ΔCT) = ΔCT_treatment_ − ΔCT_control_ (medium). Each gene’s expression was normalized, expressed as a fold increase over the control. Kruskal–Wallis test, *p* < 0.05; Dunn’s post-test: serum *vs* medium, *p < 0.05; **p < 0.01; ****p < 0.0001. The existing differences between the different groups were represented by a bar, where ^#^*p* < 0.05; ^##^*p* < 0.01; ^###^*p* < 0.001; and ^####^*p* < 0.0001. *IL6*, interleukin 6 gene **(A)**; *IL12A*, interleukin 12 alpha subunit gene **(B)**; and *TNFA*, tumor necrosis factor alpha gene **(C)**.

**Figure 2:**
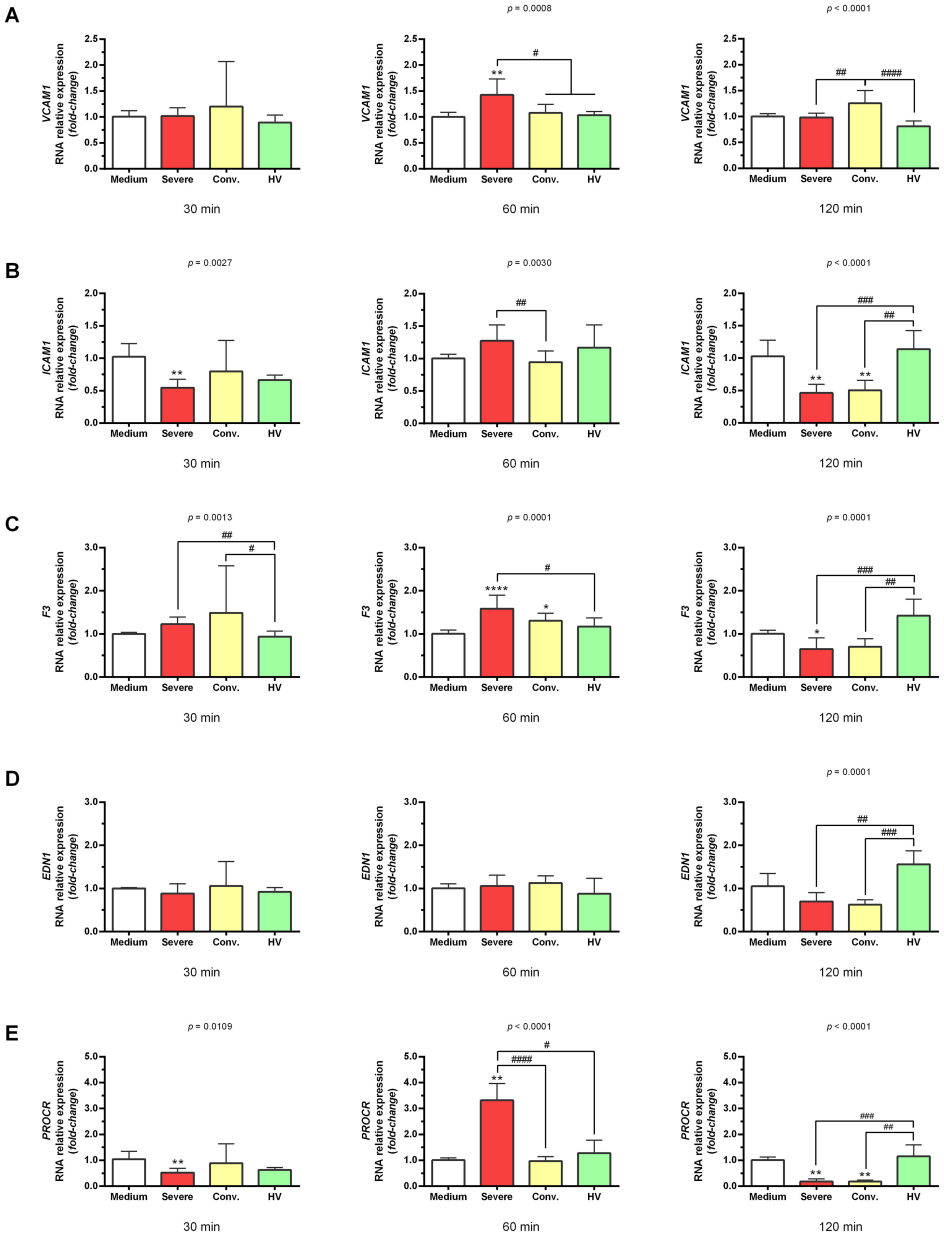
Gene expression profile related to endothelial dysfunction and thrombosis in HUVEC stimulated with serum from severe COVID-19 patients, convalescents, and healthy volunteers (HV). HUVEC were stimulated for 30 min, 60 min, and 120 min with serum from different groups: severe (n = 12), convalescent (n = 11), HV (n = 7), and culture medium (n = 6). Each value corresponds to the mean ± standard deviation. Expression values were determined by relative quantification using the following expression: Fold-change = 2^−Δ(ΔCT)^, where ΔCT = CT_target_ − CT_endogenous_ and Δ(ΔCT) = ΔCT_treatment_ − ΔCT_control_ (medium). Data are normalized as expression folded over control for each gene. Kruskal–Wallis test, *p* < 0.05; Dunn’s post-test: sera *vs* medium, *p < 0.05; **p < 0.01; ****p < 0.0001. The existing differences between the different groups were represented by a bar, where ^#^*p* < 0.05; ^##^*p* < 0.01; ^###^*p* < 0.001; and ^####^*p* < 0.0001. *VCAM1*, vascular cell adhesion molecule 1 gene **(A)**; *ICAM1*, intercellular adhesion molecule 1 gene **(B)**; *F3*, tissue factor gene **(C)**; *EDN1*, endothelin gene **(D)**; and *PROCR*, endothelial protein C receptor **(E)**.

Gene expression analysis targeting markers associated with endothelial dysfunction, thrombosis, and inflammation revealed that serum from patients with severe COVID-19 significantly upregulated the expression of *VCAM1* (1.43 ± 0.31-fold; *p <*0.01), *F3* (1.59 ± 0.31-fold; *p* < 0.0001), *PROCR* (3.32 ± 0.65-fold; *p* < 0.0001), *IL6* (1.72 ± 0.49-fold; *p <*0.01), and *IL12A* (2.26 ± 0.60-fold; *p* < 0.0001) in HUVECs after 60 min of stimulation (**Figures 1**, **2**). We highlighted the most pronounced increase in the *PROCR* gene. In contrast, serum from convalescent patients and HV did not induce these genes. When we analyzed the effect of sera from patients with severe COVID-19 on the *TNFA* gene, we noticed that there was a significant reduction in the expression of this gene (0.55 ± 0.31-fold; *p <*0.05) after 30 min of incubation, followed by an increase (1.15 ± 0.29-fold) equivalent to the negative control (medium; 1.09 ± 0.14-fold) after 60 min, and a new significant decrease (0.58 ± 0.38-fold; *p <*0.05) after 120 min (**Figure 1C**). Additionally, stimulation with these sera showed no significant changes in *EDN1* expression (**Figure 2D**).

**Figure 3:**
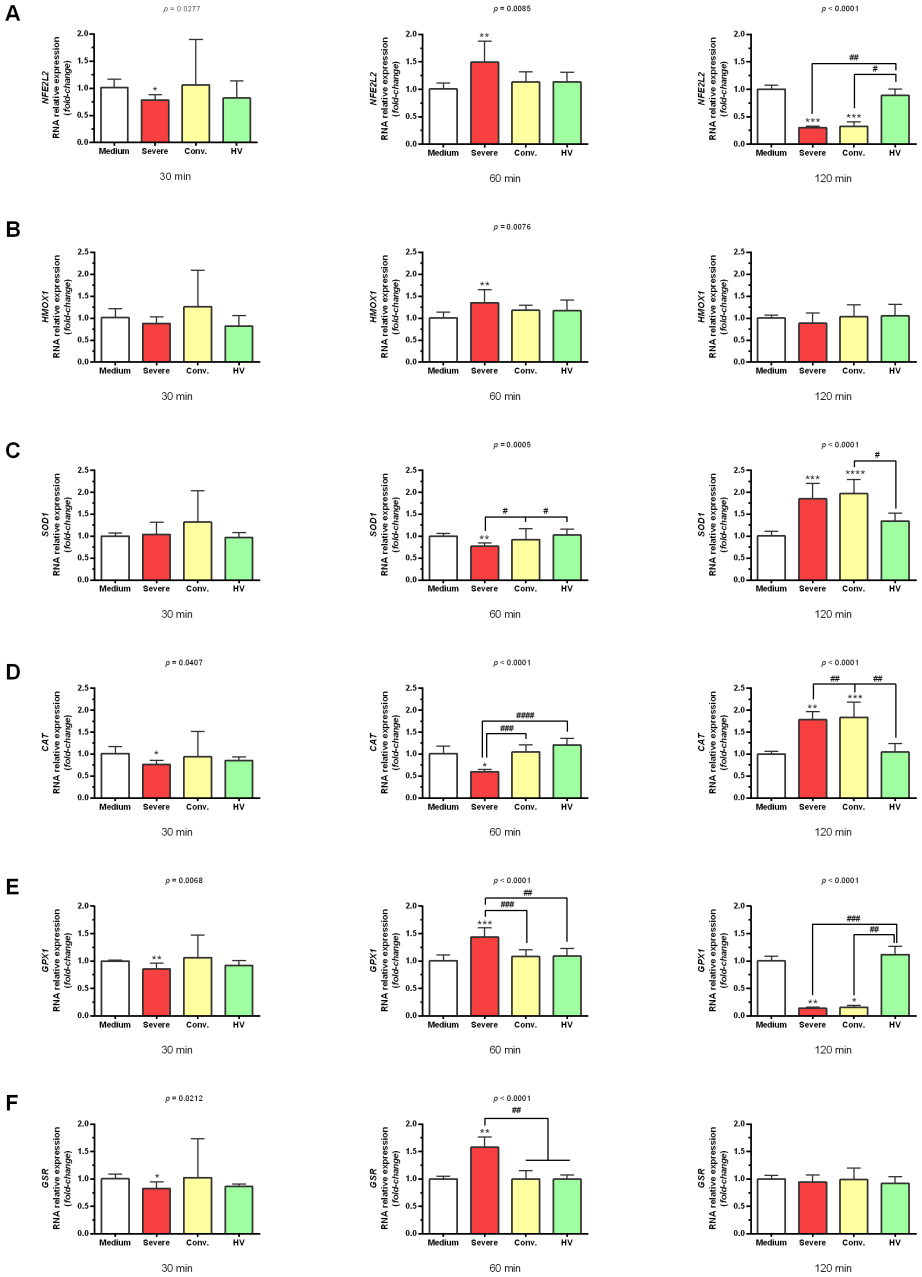
Gene expression profile related to antioxidant response in HUVEC stimulated with serum from patients with severe COVID-19, convalescents and healthy volunteers (HV). HUVEC were stimulated for 30 min, 60 min, and 120 min with serum from severe (n = 12), convalescent (n = 11), HV (n = 7), as well as with culture medium (n=6) as a control. Each value corresponds to the mean ± standard deviation. Expression values were determined by relative quantification using the following expression: Fold-change = 2^−Δ(ΔCT)^, where ΔCT = CT_target_ − CT_endogenous_ and Δ(ΔCT) = ΔCT_treatment_ − ΔCT_control (medium)_. Data are normalized as expression folded over control for each gene. Kruskal–Wallis test, *p* < 0.05; Dunn’s post-test: sera *vs* medium, *p < 0.05; **p < 0.01; ***p < 0.001; ****p < 0.0001. The existing differences between the different sera were represented by a bar, where ^#^*p* < 0.05; ^##^*p* < 0.01; ^###^*p* < 0.001; and ^####^*p* < 0.0001. *NFE2L2*, factor-related erythroid nuclear factor 2 gene **(A)**; *HMOX1*, heme oxygenase-1 gene **(B)**; *SOD1*, superoxide dismutase 1 gene **(C)**; *CAT*, catalase gene **(D)**; *GPX1*, glutathione peroxidase*1* gene **(E)**; and *GSR*, glutathione S-reductase gene **(F)**.

Concerning the genes involved in the cellular antioxidant response, serum from patients with severe COVID-19 prompted increases in *NFE2L2* (1.49 ± 0.38-fold; *p* < 0.01), *HMOX1* (1.35 ± 0.30-fold; *p* < 0.01), *GPX1* (1.44 ± 0.17-fold; *p* < 0.001), and *GSR* (1.58 ± 0.19-fold; *p* < 0.01) after 60 min of stimulation (**Figure 3**). The expression of *SOD1* and *CAT* occurred later (after 120 min) post-stimulation. Serum from severe cases resulted in increased *SOD1* (1.85 ± 0.35-fold; p <0.001) and *CAT* (1.78 ± 0.19-fold; *p <*0.0001) levels, while serum from convalescent patients induced *SOD1* and *CAT* increases of 1.97 ± 0.32-fold (*p <*0.001) and 1.83 ± 0.35-fold (*p <*0.01), respectively (**Figure 3**).

### 3.2 Serum from patients with severe COVID-19 and convalescents induce the production of markers associated with endothelial dysfunction and thrombosis in HUVEC

**Figure 4:**
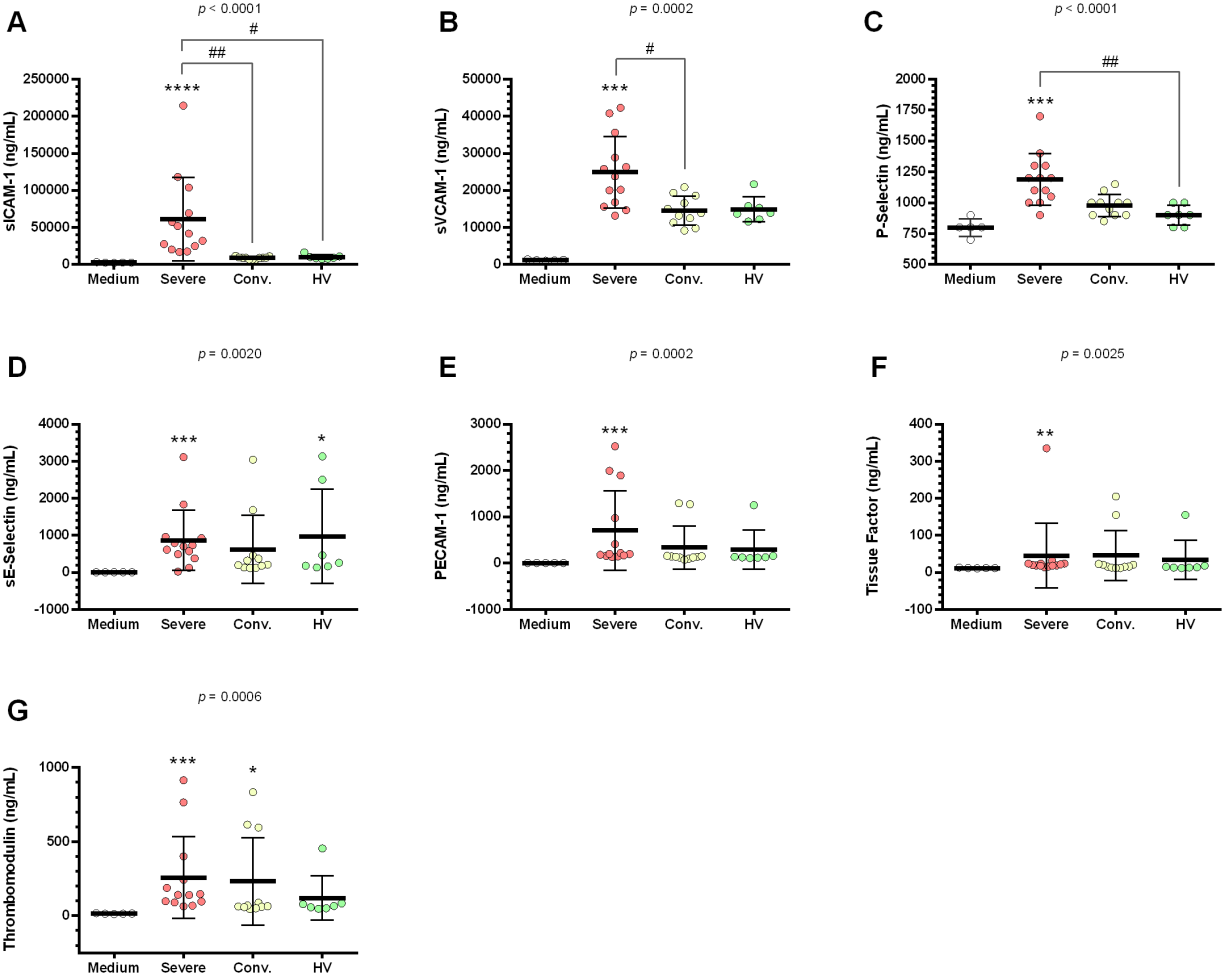
Profile of endothelial dysfunction and thrombosis markers in HUVEC supernatants stimulated with serum from severe COVID-19 patients, convalescents and healthy volunteers (HV). HUVEC were stimulated with serum from severe (n = 13), convalescent (n = 11), HV (n = 7), as well as with medium alone (n = 6) as a control. and culture medium (n = 6). This stimulation occurred over 24 h following serum deprivation. Each value corresponds to the mean ± standard deviation. Kruskal–Wallis test, *p* < 0.05; Dunn’s post-test: sera *vs* medium, *p < 0.05; **p < 0.01; ***p < 0.001; ****p < 0.0001. The existing differences between the different sera were represented by a bar, where ^#^*p* < 0.05; ^##^*p* < 0.01. sICAM-1, soluble intercellular adhesion molecule 1 **(A)**; sVCAM-1, soluble vascular cell adhesion molecule 1 **(B)**; P-selectin **(C)**; sE-Selectin, soluble E-Selectin **(D)**; PECAM-1, platelet endothelial cell adhesion molecule **(E)**; tissue factor **(F)**; and thrombomodulin **(G)**.

HUVEC supernatants stimulated with serum from patients with severe COVID-19 exhibited elevated levels of sICAM-1, sVCAM-1, P-selectin, sE-selectin, PECAM-1, tissue factor, and thrombomodulin compared to those stimulated with control medium. When contrasted with serum from HV, serum from patients with severe COVID-19 significantly increased the production of sICAM-1 in HUVEC supernatants (**Figure 4**). Serum from convalescent patients specifically enhanced thrombomodulin secretion in HUVEC (**Figure 4G**).

### 3.3 Serum from patients with severe COVID-19 and convalescents stimulate the production of pro-inflammatory cytokines and mediators in HUVEC

**Figure 5:**
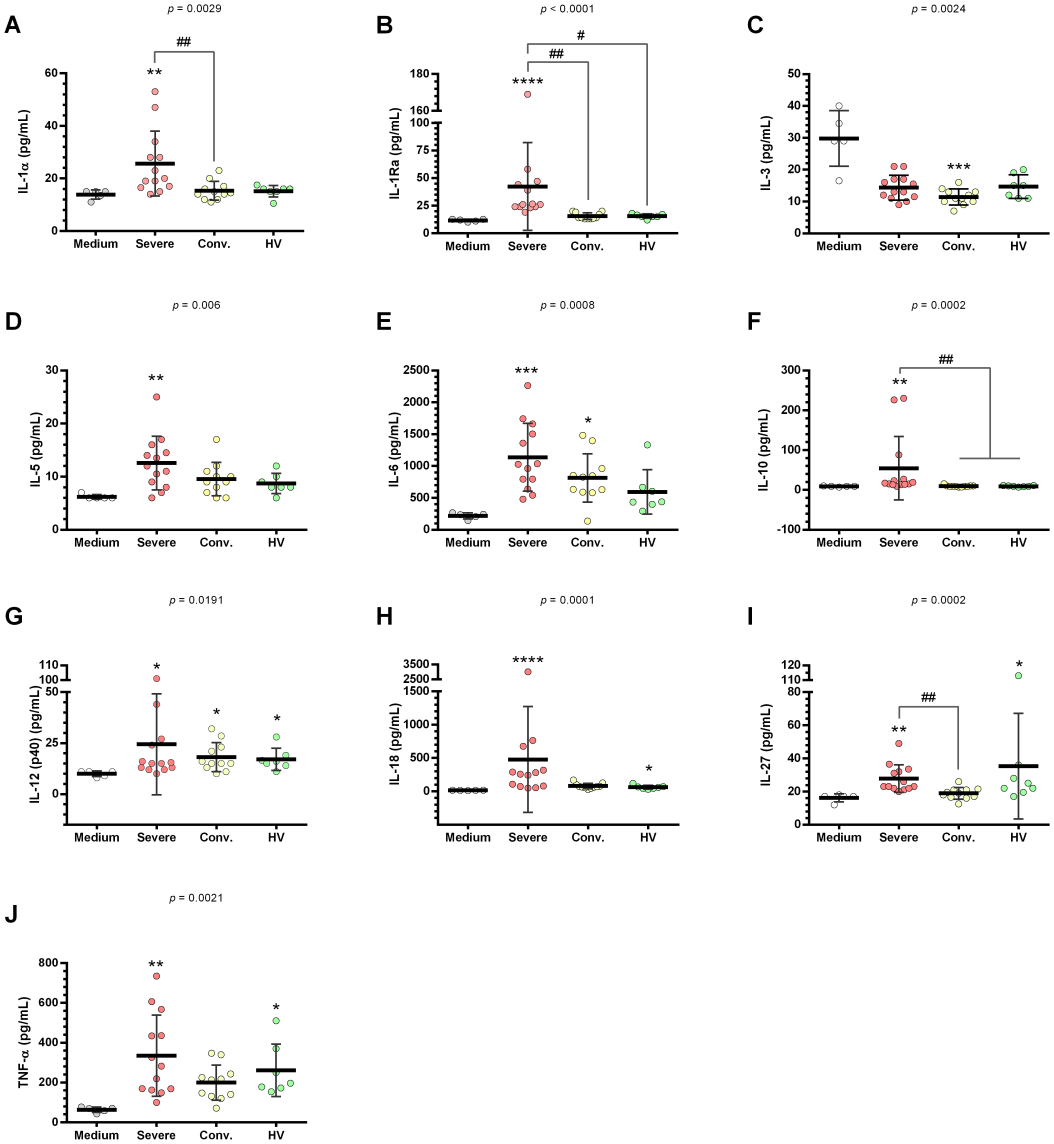
Cytokine profile in HUVEC supernatants stimulated with serum from severe COVID-19, convalescents and healthy volunteers (HV). HUVEC were stimulated with serum from severe (n = 13), convalescent (n = 11), and HV (n = 7) along with a culture medium (n = 6). This stimulation was conducted for 24 h following serum deprivation. Each value corresponds to the mean ± standard deviation. Kruskal–Wallis test, *p* < 0.05; Dunn’s post-test: sera *vs* medium, *p < 0.05; **p < 0.01; ***p < 0.001; ****p < 0.0001. The existing differences between the different sera were represented by a bar, where ^#^*p* < 0.05; ^##^*p* < 0.01. IL-1α, interleukin 1 alpha **(A)**; IL-1Ra, interleukin 1 alpha receptor antagonist **(B)**; IL-3, interleukin 3 **(C)**; IL-5, interleukin 5 **(D)**; IL-6, interleukin 6 **(E)**; IL-10, interleukin 10 **(F)**; IL-12(p40), interleukin 12 p40 subunit **(G)**; IL-18, interleukin 18 **(H)**; IL-27, interleukin 27 **(I)**; and TNF-α, tumor necrosis factor alpha **(J)**.

**Figure 6:**
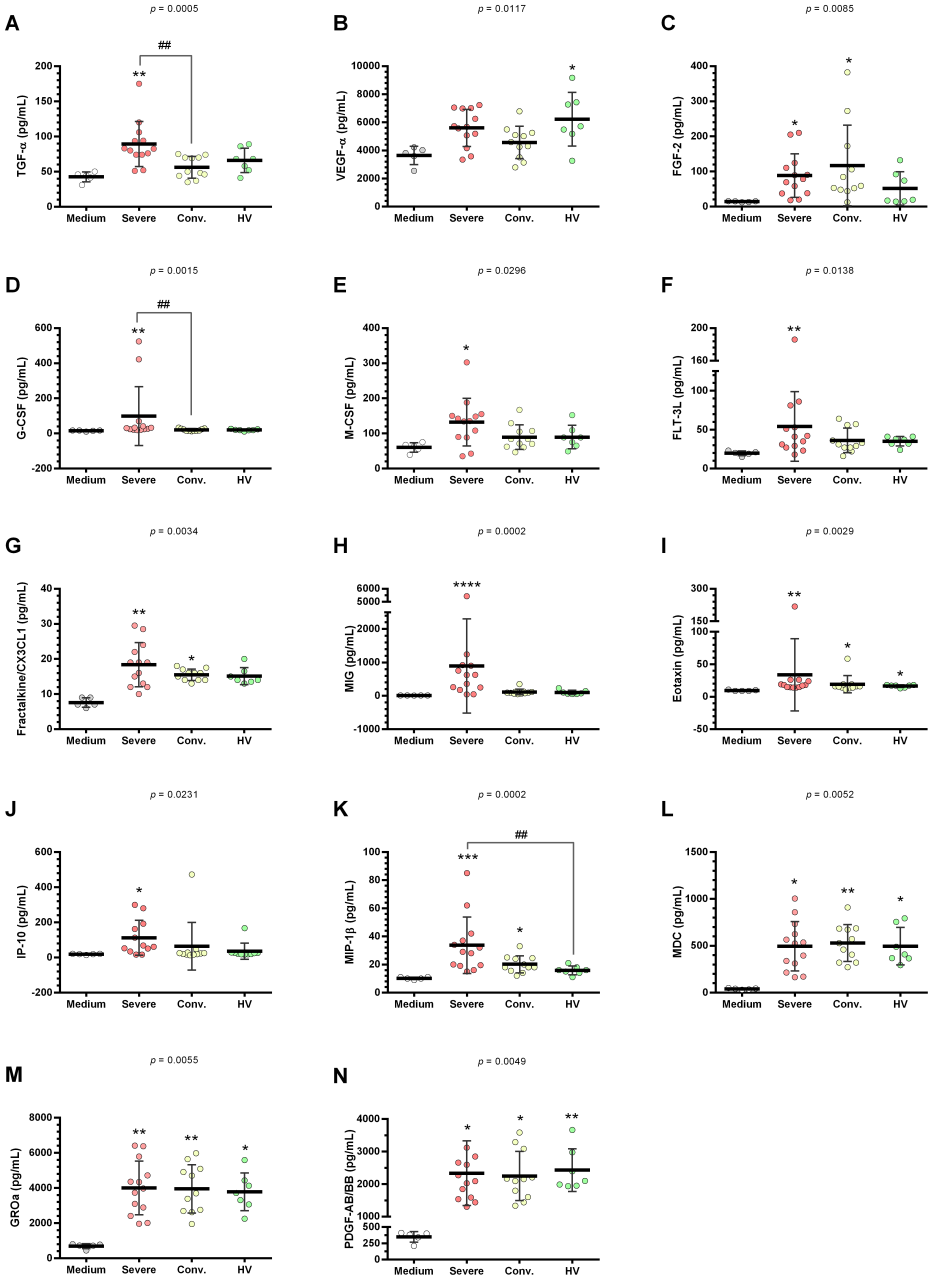
Quantification of growth factors and chemokines in HUVEC supernatants stimulated with serum from severe COVID-19 patients, convalescents and healthy volunteers (HV). HUVEC were stimulated with serum from severe (n = 13), convalescent (n = 11), and HV (n = 7), as well as culture medium (n = 6). This stimulation occurred over 24 h following serum deprivation. Each value corresponds to the mean ± standard deviation. Kruskal–Wallis test, *p* < 0.05; Dunn’s post-test: sera *vs* medium, *p < 0.05; **p < 0.01; ***p < 0.001; ****p < 0.0001. The existing differences between the different sera were represented by a bar, where ^##^*p* < 0.01. TGF-α, Transforming growth factor alpha **(A)**; VEGF-β, vascular endothelial growth factor B **(B)**; FGF-2 (or bFGF), basic fibroblast growth factor **(C)**; G-CSF, granulocyte colony stimulating factor **(D)**; M-CSF, monocyte colony stimulating factor **(E)**; FLT-3L, FMS-related tyrosine kinase 3 ligand **(F)**; fractalkine (CX3CL1) **(G)**; MIG, interferon gamma-induced monokine **(H)**; eotaxin **(I)**; IP-10, interferon gamma-induced protein 10 **(J)**; MIP-1β, macrophage inflammatory protein-1 beta **(K)**; MDC, macrophage-derived chemokine **(L)**; GROa (or CXCL1), growth-regulated alpha protein **(M)**; and PDGF-AB/BB, platelet-derived growth factor-AB/BB **(N)**.

HUVEC supernatants, when stimulated with serum from patients with severe COVID-19 compared to control medium, showed a marked increase in a range of cytokines including IL-1α, IL-1Ra, IL-5, IL-6, IL-10, IL-12(p40), IL-18, IL-27, TNF-α, TGF-α, FGF-2, G-CSF, M-CSF, FLT-3L, fractalkine, MIG, eotaxin, IP-10, MIP-1β, MDC, and GROa (**Figures 5**, **6**).

In contrast, HUVEC supernatants stimulated with serum from convalescent patients compared to the control medium, exhibited an increase in a more limited set of cytokines, specifically IL-12(p40), IL-18, FGF-2, eotaxin, MIP-1β, MDC, and GROa (**Figures 5**, **6**). Furthermore, stimulation with convalescent serum led to a significant reduction in IL-3 levels (**Figure 5**).

When comparing HUVEC supernatants stimulated with serum from HV to the control medium, there was a significant increase in the levels of IL-12(p40), IL-18, IL-27, TNF-α, VEGF, eotaxin, MDC, GROa, and PDGF-AB/BB (**Figures 5**, **6**). The results for the other cytokines, growth factors, and chemokines that did not show significant changes are detailed in **Supplementary Figure 1**.

Furthermore, serum from patients with severe COVID-19 significantly stimulated the production of IL-1ra and IL-10 compared to HV. Elevated levels of MIP-1b levels were also observed in HUVEC supernatants after stimulation with serum from severe COVID-19 cases compared to HV. Moreover, serum from convalescent patients demonstrated reduced production of TGF-α and G-CSF compared with that from patients with severe COVID-19. Notably, convalescent serum showed significant decreases in sICAM-1, sVCAM-1, P-selectin, IL-1α, IL-1ra, IL-10, IL-27, TNF-α, TGF-α, and G-CSF compared to serum from severe COVID-19 patients.

### 3.4 Assessing biomarker efficacy in distinguishing health conditions

**Figure 7:**
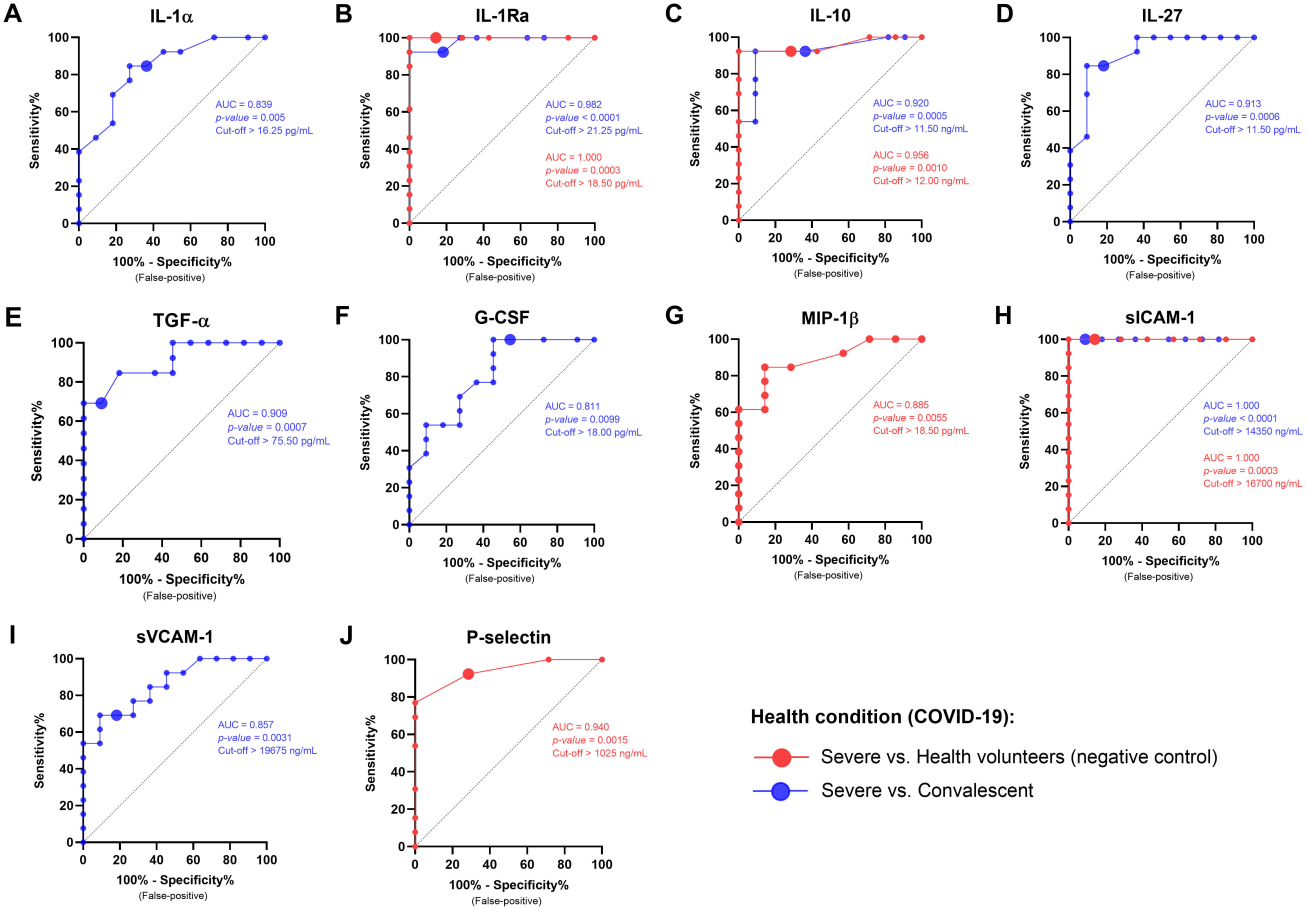
ROC curve analysis of biomarkers in HUVEC stimulated by serum from patients with different health conditions (COVID-19). The graphs show the sensitivity and specificity of markers that have statistical significance of patients with severe COVID-19 *vs*. HV and severe COVID-19 *vs*. HV. AUC, Area under the curve; CI, confidence interval. IL-1α, interleukin 1 alpha **(A)**; IL-1Ra, interleukin 1 alpha receptor antagonist **(B)**; IL-10, interleukin 10 **(C)**; IL-27, interleukin 27 **(D)**; TGF-α, transforming growth factor alpha **(E)**; G-CSF, granulocyte colony stimulating factor **(F)**; MIP-1β, macrophage inflammatory protein-1 beta **(G)**; sICAM-1, soluble intercellular adhesion molecule 1 **(H)**; sVCAM-1, soluble vascular cell adhesion molecule 1 **(I)**; and P-selectin **(J)**.

ROC curve analysis was conducted to assess the sensitivity and specificity of biomarker concentrations in discriminating health conditions. Comparing the severe group and HV, the analyses demonstrated statistically significant curves for IL-1Ra (AUC = 1.000; cut off >18.50 pg/mL), IL-10 (AUC = 0.956; cut off >12.00 pg/mL), sICAM-1 (AUC = 1.000; cut off >16,700 ng/mL), MIP-1β (AUC = 0.885; cut off >18.50 pg/mL), and P-selectin (AUC = 0.940; cut off >1,025 ng/mL). Comparisons between the severe and convalescent groups also showed significant curves for IL-1α (AUC = 0.839; cut off >16.25 pg/mL), IL-1Ra (AUC = 0.982; cut off >21.50 pg/mL), IL-10 (AUC = 0.920; cut off >11.50 pg/mL), IL-27 (AUC = 0.913; cut off >21.75 pg/mL), TGF-α (AUC = 0.909; cut off >75.50 pg/mL), G-CSF (AUC = 0.811; cut off >18.00 pg/mL), sICAM-1 (AUC = 1.000; cut off >14,350 ng/mL), and sVCAM-1 (AUC = 0.857; cut off >19,675 ng/mL) (**Figure 7**; **Supplementary Table 1**).

**Table 1** presents the MLR data. The proposed model significantly predicted health conditions (*F* (5, 19) = 8.947, *p* = 0.001). The adjusted R^2^ of 0.507 indicates that the model accounts for 50.7% of the variance in health conditions. Notably, reduced IL-10 levels were significantly associated with severe disease (β = −1.130), whereas increased levels of MIP-1β, sICAM-1, and P-selectin were strongly linked to severe disease (β = 1.145, 0.724, and 0.464, respectively).

**Table 1:** Multivariate linear regression model comparing HUVECs stimulated by serum from patients with different health conditions (COVID-19).

Dependent variable	Independent variable	Adjusted R^2^	β	*t-value*	*F-test*	*p_1_*	*p_2_*
Health condition*	IL-1Ra	0.677	−0.158	−0.656	8.947	**0.001**	0.522
	IL-10		−1.130	−3.499			**0.004**
	MIP-1β		1.145	3.166			**0.007**
	sICAM-1		0.724	3.635			**0.003**
	P-selectin		0.464	3.273			**0.006**

R^2^, coefficient of determination; β, coefficient of regression; *p_1_*, *p*-value of the model; *p_2_*, *p*-value of the independent variable. The final predictive equations of the dependent variables were Health condition = [(−0.158 × IL-1Ra concentration) + (−1.130 × IL-10 concentration) + (1.145 × MIP-1β concentration) + (0.724 × sICAM-1 concentration) + (0.464 × P-selectin concentration)] − 1.207; * Health condition was categorized as 0: health volunteer and 1: individuals with severe COVID-19.*p*-Values with statistical significance are highlighted in bold.

## 4 Discussion

The findings indicate that HUVEC exhibit distinct responses when stimulated with serum from patients with severe COVID-19, convalescents, and HV, demonstrating the endothelium’s critical role in the body’s reaction to COVID-19. Notably, serum from patients with severe COVID-19 induced significant upregulation of genes associated with endothelial dysfunction, thrombosis, inflammation, and antioxidant responses in HUVECs. This suggests robust endothelial activation and dysfunction in severe COVID-19 cases, which could contribute to the increased thromboembolic and inflammatory complications observed in these patients.

Initially, we assessed the expression of *IL6*, *IL12A*, and *TNFA*, which are key pro-inflammatory genes implicated in the cytokine storm associated with COVID-19. Our results revealed that serum from patients with severe COVID-19 significantly upregulated *IL6* and *IL12A* expression in HUVEC within 60 min of stimulation.

Although there was no significant increase in *TNFA* expression at this early stage, we noted a marked decrease in its expression 120 min after stimulation with serum from both severe and convalescent patients compared to that from HV. Additionally, substantial reductions were observed in the expression of *IL6* and *IL12A* at later time points. This rapid decline may be attributed to the swift synthesis and subsequent conversion of mRNA into protein products, illustrating that the serum from patients induces a distinctly different response in HUVEC than that induced by serum from HV. The increase in IL-6, IL-12, and TNF-α levels in HUVEC supernatants further supports our hypothesis regarding the dynamic regulation of inflammatory mediators in response to COVID-19.

Serum from patients with severe COVID-19 significantly upregulated genes involved in the cellular antioxidant response in HUVEC, including *NFE2L2*, *HMOX1*, *SOD1*, *CAT*, *GPX1*, and *GSR*. The later expression of *SOD1* and *CAT* after 120 min could indicate a delayed but essential antioxidant defense mechanism against oxidative stress induced by severe inflammation. NFE2L2 (also known as NRF2) plays a pivotal role in the cytoprotective response to oxidative stress, although the mechanisms by which it combats SARS-CoV-2 infection remain poorly understood (33).

Genetic variation in *NFE2L2* and *KEAP1* may influence acute lung injury by modifying the activation of this protective pathway, potentially accounting for varying susceptibilities to different clinical stages of SARS-CoV-2 infection (32). This highlights the critical importance of maintaining NFE2L2 integrity to trigger antioxidant enzyme induction, providing a cytoprotective response against pro-oxidant insults from the virus.

Catalase is widely distributed in epithelial cells and leukocytes, among others, and protects them against oxidative damage, regulates cytokine production, and slows the replication of SARS-CoV-2 (34). Mo et al. (35) showed that enhancing SOD production could prevent plasma cell apoptosis upon re-exposure and dramatically prolong SARS-CoV-specific antibody production. Another study showed that serum concentrations of SOD and GPx were significantly higher in patients with COVID-19 than in controls, indicating an activated antioxidant response (36). Conversely, significantly lower GSR and elevated levels of IL-10 in COVID-19 patients suggest deficiencies in antioxidant capabilities and immune function, respectively. Our data indicated a distinct response in HUVEC, with an increase in GSR expression. Supporting this finding, Naghashpour et al. (37) showed that serum from patients with severe COVID-19 strongly induced *GSR* expression, underscoring the potential role of enhanced antioxidant responses in severe disease stages.

The elevated expression of genes such as *VCAM1*, *F3*, *PROCR*, *IL6*, and *IL12A* within 60 min of stimulation indicates an acute and rapid endothelial response to the severe inflammatory state induced by COVID-19 infection. The lack of gene induction by serum from convalescent patients and healthy volunteers underscores the unique pathological environment presented by severe COVID-19, which appears to persist beyond the acute phase of disease.

The production of soluble markers, such as sICAM-1, sVCAM-1, P-selectin, sE-selectin, PECAM-1, tissue factor, and thrombomodulin, further illustrates extensive endothelial activation and the potential for increased vascular permeability and thrombosis. This is particularly notable in severe cases compared to convalescent patients, where only thrombomodulin showed a significant increase.

Vascular cell adhesion molecule-1 (VCAM-1) and ICAM-1 levels were found to be elevated in the blood of patients with mild COVID-19 and dramatically elevated in severe cases, decreasing during the convalescence phase (38). This trend may explain the increased expression of the corresponding genes in the present study. Pro-inflammatory cytokines, such as IL-6, IL-1β, and TNF-α, are known to induce *F3* gene expression in both immune and non-immune cells, potentially contributing to the thromboembolic events observed in COVID-19 patients, particularly in severe cases (39). Furthermore, a meta-analysis showed that tissue factor levels were positively associated with senescence and hypercoagulation gene signatures in patients with COVID-19. This study underscores the role of tissue factors as a critical link between inflammation, thrombosis, and senescence in respiratory viral infection (40).

The distinct cytokine profile, characterized by elevated levels of pro-inflammatory cytokines in HUVECs supernatants stimulated by serum from severe COVID-19 patients, suggests a hyperactive inflammatory response that could exacerbate endothelial injury and dysfunction. This hyperactivity contrasts with the more restrained cytokine production observed in HUVECs stimulated with serum from convalescent patients, indicating a gradual resolution of the inflammatory response.

HUVEC substantially express the *PROCR* gene, which encodes the endothelial protein C receptor (EPCR). EPCR is associated with anticoagulant and anti-inflammatory roles, regulating hemostasis and inflammation (41). Some studies have reported that mutations in the *PROCR* gene are associated with the development of thrombosis (42, 43). EPCR is an important controller of the protein C anticoagulant pathway, binding to protein C and potentiating its activation by the thrombomodulin-thrombin complex. EPCR binds to protein C and activated protein C (APC) with high affinity.

A recent study by Won et al. (44) showed that pulmonary endothelial cells from patients who died of severe COVID-19 had increased expression of von Willebrand procoagulant factor (VWF) and decreased expression of the anticoagulant thrombomodulin and EPCR. Although we did not measure EPCR in the culture supernatant, the remarkable increase in *PROCR* gene expression suggests that critically ill patients respond by inducing the antithrombotic system to compensate for the thrombogenic condition in COVID-19.

We then assessed the cytokine levels in HUVEC supernatants stimulated with sera. Serum from patients with severe COVID-19 induced increased levels of IL-1α, IL-1Ra, IL-5, IL-6, IL-10, IL-12(p40), IL-18, IL-27, and TNF-α.

Previous reports have shown that the pathogenesis of COVID-19 is supported by a potent inflammatory response involving various mediators, such as IL-6 and IL-10. These pleiotropic cytokines are produced at inflammation sites by different cell types, including lymphocytes, endothelial cells, and epithelial cells, and are released into the circulation during sepsis and acute organ injury (45). Furthermore, IL-6, IL-12, and TNF-α are cytokines classically known to have pro-inflammatory properties and are elevated in COVID-19 (46). Studies carried out with sera from patients with various degrees of severity of COVID-19 showed that patients had higher levels of these markers (47, 48). Our results corroborate these findings, suggesting that endothelial cells may contribute to the production of these cytokines in response to stimulation of sera from patients infected with SARS-CoV-2 in severe conditions. Previous studies have shown that endothelial cells can produce cytokines after infection (49, 50).

Although none of the sera showed a significant increase in *TNFA* expression, the results of assaying HUVEC supernatants showed that sera from patients with severe COVID-19 significantly induced the production of TNF-α. Based on this information, it is possible to suggest that the expression of the *TNFA* gene may have occurred shortly after the stimuli, with subsequent production of the cytokine.

Shao et al. (51) showed that Human Mammary Epithelial Cells (HMEC) have trained immunity in response to subsequent MERS-CoV-2 infections, which contributes to upregulation of pro-inflammatory cytokines genes, such as *TNFA*, *IL6*, *CSF1*, *CSF3* and *IL32* (trained immunity marker), and indicates that trained immunity in infected HMEC can generate cytokine storms.

Elevated levels of IL-1α were associated with lung injury in patients with severe COVID-19 (52). In contrast, the IL-1Ra is a member of the IL-1 cytokine family and is secreted by various cell types, including epithelial cells, immune cells, hepatocytes, keratinocytes, and fibroblasts. It acts as a natural inhibitor of the proinflammatory effect of IL-1β (53) and is significantly associated with the severity and progression of COVID-19 (24).

Han et al. (45) showed that high levels of cytokine storms are directly associated with disease severity and are associated with IL-6, suggesting that these markers can be used as predictors for the rapid diagnosis of patients at higher risk of disease severity. In contrast, IL-10 is a Th2-type cytokine that exhibits anti-inflammatory action against cytokines such as IL-6, IL-1β, IFN-γ, and TNF-α in different cell types, preventing the maturation of dendritic cells by blocking the production of IL-12 (54).

IL-18 is involved in the activation of Th1 and natural killer cells, being able to induce IFN-γ in the presence of IL-12 or IL-15. IL-18 acts on CD4, CD8, and NK cells to induce IFN-γ production (55). Furthermore, macrophages can produce IFN-γ when activated by IL-18 and IL-12 (56).

IL-27 has a dual role, being able to stimulate the differentiation of Th1 cells that express IFN-γ, as well as IL-10-producing T cells (57). IL-27 acts on several cell types, including T and B cells, macrophages, dendritic cells, NK cells, and non-hematopoietic cells (58). The expansion of Th1 cells occurs through the transcription-mediated T-bet signaling pathway (STAT) 1 (59). In contrast, IL-27 also acts by suppressing immune responses through the inhibition of Th17 cell development and induction of IL-10 production in a STAT1- and STAT3-dependent manner (60, 61). Circulating IL-27 levels are positively correlated with severity and worse prognosis in patients with severe COVID-19 (62). Xu et al. (63) showed in a recent study that serum IL-27 levels are positively associated with severity and poor prognosis in patients with pneumonia, indicating that IL-27 may be involved in the pathophysiological process of the disease. Therefore, these data suggest that IL-27 may be involved in the pathogenesis of inflammatory lung diseases.

In individuals infected with SARS-CoV-2, Th1 and Th2 cytokines are elevated, Wolf et al. (64) reported a consistently elevated expression of type 1 interferons, Th1 and Th2 interleukins, and chemokines early in SARS-CoV-2 infection. The observed increased IL-5 levels corroborate the findings of Montazersaheb et al. (65) who reported a correlation between the severity of COVID-19 and the reduction in T cells. This leads to the overexpression of Th2-derived cytokines such as IL-4, IL-5, and IL-13 (65, 66).

Previous studies have shown that FGF2 is involved in the strong apoptotic response induced by MERS-CoV, which is fundamental for the development of increased replication by the lytic cycle in the lungs (67). Furthermore, FGF2 plays a role in intussusceptive angiogenesis and induces angiogenesis due to the marked inflammatory process and hypoxia that occur in the disease (68). Meini et al. (69) suggest that intussusceptive angiogenesis may have the function of restoring a functional vascular plexus that results from extensive endothelialitis and alveolar capillary microthrombosis in COVID-19.

Fractalkine (CX3CL1) is a chemokine produced by the activated endothelium in response to stimuli with pro-inflammatory cytokines, such as TNF-α and IL-1β (70, 71), and acts as an important regulator of endothelial function and dysfunction, including thrombosis (72). CX3CL1 may be upregulated in the endothelium during SARS-CoV-2 infection and contribute to the perpetuation of a prothrombotic cycle (72).

MIG (CXCL9) and IP-10 (CXCL10) are involved in Th1, CD8, and NK cell migration and early upregulation after SARS-CoV-2 infection (73). Higher MIG levels are detected in mildly symptomatic and severe patients than in asymptomatic patients (74). IP-10 is produced by activated bronchial and alveolar epithelial cells and is implicated in apoptosis and the development of lymphopenia and neutrophilic infiltration, and its levels increase with disease severity or death (74).

Studies have shown that patients who died with COVID-19 had elevated plasma levels of CX3CL1, CXCL9, and CXCL10, among others, compared to patients with severe and/or mild COVID-19 (73).

The Flt-3L ligand is a hematopoietic cytokine that stimulates the proliferation and differentiation of several blood cell progenitors and is an important growth factor that stimulates the growth of dendritic cells (75).

In summary, serum from patients with severe COVID-19 significantly induced the expression of genes associated with endothelial dysfunction, thrombosis, inflammation, and oxidative stress. Conversely, serum from convalescents exhibited an expression profile similar to that of healthy volunteers, with no notable increases in target gene expression at the evaluated times, except for a discrete yet significant induction of the tissue factor gene.

Additionally, HUVEC supernatants stimulated with serum from severe cases showed a substantially higher presence of cytokines, chemokines, and inflammation-related growth factors than those from convalescents and healthy volunteers.

Specifically, P-selectin and MIP-1β levels were elevated in HUVEC supernatants stimulated with serum from patients with severe COVID-19 compared to those from HV. Comparing the severe and convalescent groups, we observed an observed increase in IL-27, TGF-α, sVCAM-1, IL-1α, and G-CSF. Analysis of the ROC curve results revealed common markers between patients with severe COVID-19 and both HV and convalescent groups, including IL-1Ra, IL-10, and sICAM-1, as diagnostic tools to distinguish between health conditions with high sensitivity and specificity.

Moreover, MLR analysis reinforced the significance of IL-10, MIP-1 β, s-ICAM-1, and P-selectin as indicators of disease severity. Decreased IL-10 and increased MIP-1β, sICAM-1, and P-selectin levels were associated with greater disease severity, emphasizing the critical importance of these markers in understanding and managing COVID-19.

These findings suggest that these biomarkers can be useful for distinguishing the health status of critically ill and convalescent patients, underscoring the significant role of the endothelium in the pathophysiology of COVID-19. This role persists even in convalescent individuals, as SARS-CoV-2 infection can lead to long-term complications. However, additional research is required to substantiate this hypothesis.

## 5 Conclusion

Based on our findings, HUVEC demonstrates significant potential as biological sensors in response to serum stimuli from patients with COVID-19. These cells contribute to the production of cytokines, chemokines, and growth factors, which are crucial in the inflammatory response, oxidative stress, and procoagulant state observed in response to inflammatory mediators present in the serum of patients with the disease. This highlights the critical role of the endothelium in the pathophysiology of COVID-19, underscoring its importance in both disease progression and potential therapeutic targeting.

## Data Availability

The original contributions presented in the study are included in the article/**Supplementary Material**. Further inquiries can be directed to the corresponding author.
