# Supplementary material for: Assessing the endothelium’s role in COVID-19 severity using the HUVEC model
Source: Front Immunol. 2026 Jan 8;16:1689772. doi: 10.3389/fimmu.2025.1689772 (PMC12823526; doi:10.3389/fimmu.2025.1689772)
Supplement: Supplementary file 1 [file DataSheet1.docx]

Supplementary Material

**

**

**Supplementary Figure 1.** **Measurement of growth factors and cytokines in supernatants of HUVEC stimulated with sera from severe COVID-19 patients, convalescents and controls that did not show statistical significance.** HUVEC were stimulated with severe (n=13), convalescent (n=11) and HV sera (n=7) and medium (n=6) for 24 h after deprivation.

| **Supplementary Table 1. The ROC curve analysis was conducted to assess the sensitivity and specificity of biomarker concentrations in discriminating health conditions** | | | | | | | |
| --- | --- | --- | --- | --- | --- | --- | --- |
| Markers | AUC^a^  (95% CI) | Std. Error^b^ | *P*-value^c^ | Sensitivity (%)  (95% CI) | Specificity (%)  (95% CI) | Likelihood Ratio | Cut off |
| *Severe vs. Health Volunteer* | | | | | | | |
| IL-1Ra | 1.000  (1.000 – 1.000) | 0.000 | 0.0003 | 100.00  (77.19 – 100.00) | 100.00  (64.57 – 100.00) | ---- | > 18.50 pg/mL |
| IL-10 | 0.956  (0.865 – 1.000) | 0.047 | 0.0010 | 92.31  (66.69 – 99.61) | 100.00  (64.57 – 100.00) | ---- | > 12.00 pg/mL |
| sICAM-1 | 1.000  (1.000 – 1.000) | 0.000 | 0.0003 | 100.00  (77.19 – 100.00) | 100.00  (64.57 – 100.00) | ---- | > 16700 ng/mL |
| MIP-1β | 0.885  (0.737 – 1.000) | 0.075 | 0.0055 | 84.62  (57.77 – 97.27) | 85.71  (48.69 – 99.27) | 5.923 | > 18.50 pg/mL |
| P-selectin | 0.940  (0.840 – 1.000) | 0.051 | 0.0015 | 76.92  (49.74 – 91.82) | 100.00  (64.57 – 100.00) | ---- | > 1025 ng/mL |
| *Severe vs. Convalescent* | | | | | | | |
| IL-1α | 0.839  (0.680 – 0.998) | 0.081 | 0.0050 | 84.62  (57.77 – 97.27) | 72.73  (43.44 – 90.25) | 3.103 | > 16.25 pg/mL |
| IL-1Ra | 0.982  (0.940 – 1.000) | 0.215 | < 0.0001 | 92.31  (66.69 – 99.61) | 100.00   1. – 100.00) | ---- | > 21.50 pg/mL |
| IL-10 | 0.920  (0.803 – 1.000) | 0.060 | 0.0005 | 92.31  (66.69 –99.61) | 90.91  (62.26 – 99.53) | 10.150 | > 11.50 pg/mL |
| IL-27 | 0.913  (0.794 – 1.000) | 0.061 | 0.0006 | 84.62  (57.77 –97.27) | 90.91  (62.26 –99.53) | 9.308 | > 21.75 pg/mL |
| TGF-α | 0.909  (0.794 – 1.000) | 0.059 | 0.0007 | 69.23  (42.37 – 87.32) | 100.00  (74.12 – 100.00) | ---- | > 75.50 pg/mL |
| G-CSF | 0.811  (0.638 – 0.9849) | 0.886 | 0.0099 | 100.00  (77.19 – 100.00) | 54.55  (28.01 – 78.73) | 2.200 | > 18.00 pg/mL |
| sICAM-1 | 1.000  (1.000 – 1.000) | 0.000 | < 0.0001 | 100.00  (77.19 – 100.00) | 100.00  (74.12 – 100.00) | ---- | > 14350 ng/mL |
| sVCAM-1 | 0.857  (0.710 – 1.000) | 0.075 | 0.0031 | 69.23  (42.37 – 87.32) | 90.91  (62.26 – 99.53) | 7.615 | > 19675 ng/mL |
| a. Area Under the Curve; b. Under the nonparametric assumption; c. Null hypothesis: true area = 0.5. | | | | | | | |
